# Longitudinal *in vivo* Diffusion Tensor Imaging Detects Differential Microstructural Alterations in the Hippocampus of Chronic Social Defeat Stress-Susceptible and Resilient Mice

**DOI:** 10.3389/fnins.2018.00613

**Published:** 2018-08-29

**Authors:** Xiao Liu, Jizhen Yuan, Yu Guang, Xiaoxia Wang, Zhengzhi Feng

**Affiliations:** ^1^School of Psychology, Army Medical University, Chongqing, China; ^2^Department of Microbiology, College of Basic Medical Sciences, Army Medical University, Chongqing, China

**Keywords:** chronic social defeat stress, susceptibility, resilience, dorsoventral hippocampus, diffusion tensor imaging (DTI), longitudinal

## Abstract

**Background:** Microstructural alterations in the hippocampus may underlie stress-related disorders and stress susceptibility. However, whether these alterations are pre-existing stress vulnerability biomarkers or accumulative results of chronic stress remain unclear. Moreover, examining the whole hippocampus as one unit and ignoring the possibility of a lateralized effect of stress may mask some stress effects and contribute to the heterogeneity of previous findings.

**Methods:** After C57BL/6 mice were exposed to a 10-day chronic social defeat stress (CSDS) paradigm, different stress phenotypes, i.e., susceptible (*n* = 10) and resilient (*n* = 7) mice, were discriminated by the behavior of the mice in a social interaction test. With *in vivo* diffusion tensor imaging (DTI) scans that were conducted both before and after the stress paradigm, we evaluated diffusion properties in the left and right, dorsal (dHi) and ventral hippocampus (vHi) of experimental mice.

**Results:** A significantly lower fractional anisotropy (FA) was found in the right vHi of the susceptible mice prior to the CSDS paradigm than that found in the resilient mice, suggesting that pre-existing microstructural abnormalities may result in stress susceptibility. However, no significant group differences were found in the post-stress FA values of any of the hippocampal regions of interest (ROIs). In addition, mean diffusivity (MD) and radial diffusivity (RD) values were found to be significantly greater only in the right dHi of the resilient group compared to those of the susceptible mice. Furthermore, a significant longitudinal decrease was only observed in the right dHi RD value of the susceptible mice. Moreover, the social interaction (SI) ratio was positively related to post-stress left MD, right dHi MD, and right dHi RD values and the longitudinal right dHi MD percent change. Meanwhile, a negative relationship was detected between the SI ratio and bilateral mean of the post-stress left relative to right vHi FA value, highlighting the important role of right hippocampus in stress-resilience phenotype.

**Conclusion:** Our findings demonstrated different longitudinal microstructural alterations in the bilateral dHi and vHi between stress-susceptible and resilient subgroups and indicated a right-sided lateralized stress effect, which may be useful in the diagnosis and prevention of stress-related disorders as well as their intervention.

## Introduction

Chronic stress contributes to several serious psychiatric disorders, including depression and post-traumatic stress disorder (PTSD) (Heim et al., 2008; Muscatell et al., 2009; Hunter et al., 2010; Pizzagalli, 2014), in which the hippocampus plays a critical regulatory role and its structure and function are affected (Smith, 2005; Amico et al., 2011; Delgado y Palacios et al., 2011; Zannas et al., 2013; Tse et al., 2014; O’Doherty et al., 2015; Otten and Meeter, 2015; Chan et al., 2016; Morey et al., 2016). The alterations in hippocampal morphology, such as changes in neuronal numbers, dendritic length and neurite density, are known to be associated with behavioral changes under stress (Czeh et al., 2006; Li et al., 2011, 2014; Yang et al., 2015; Gilabert-Juan et al., 2017). However, due to the paucity of longitudinal studies, whether the microstructural changes in the hippocampus pre-exist in stress-vulnerable individuals as a susceptible biomarker or are accumulative results caused by persisting stress exposure is still unclear (Sheline, 2011). Determining the direction of this confounding causality will be helpful for elaborating the mechanism that underlies stress-related disorders and for developing more customized interventions.

As a non-invasive imaging method, diffusion tensor imaging (DTI) can be repeatedly employed in longitudinal studies and is able to sensitively detect microstructural organization by measuring physical properties of water molecule diffusion in the tissue of interest (Alexander et al., 2007; Kumar et al., 2014). Among the most widely used diffusion indices, fractional anisotropy (FA) reflects the degree of tissue integrity and alignment of cellular structure (Beppu et al., 2005; Kinoshita et al., 2008; Anacker et al., 2016), while mean diffusivity (MD) is more related to cell density and size (Kumar et al., 2014). In addition, the radial diffusivity (RD) and axial diffusivity (AD) should be considered in the interpretation of the FA value, for they represent myelin and axonal integrity, respectively, and pathological alterations in either can lead to a decrease in FA. A combination of these four indices may represent a comprehensive understanding of the microstructural changes in the hippocampus under stress.

For longitudinal studies, given the limitations of clinical studies (Nestler and Hyman, 2010), various animal models of stress have been employed as substitutes to establish a better understanding of stress-related disorders. Among those, the chronic social defeat stress (CSDS) model mimics a psychosocial stressor in real life and not only is of great construct, face, discriminative and predictive validity but also consistently generates susceptible and resilient subgroups characterized by the presence or absence of social withdrawal in a post-stress social interaction test, respectively (Golden et al., 2011). Stress resilience, which describes when people “achieve a positive outcome in the face of adversity” (McEwen et al., 2015), has been demonstrated to be due to an active stress coping mechanism rather than simply a passive absence of stress-induced pathophysiological changes (Charney, 2004; Krishnan et al., 2007; Russo et al., 2012; Friedman et al., 2014). In addition, based on the CSDS model, previous studies have demonstrated distinctions between the two stress phenotypes from various perspectives, such as gene expression (Krishnan et al., 2007), protein expression (Carboni et al., 2006; Stelzhammer et al., 2015), and neurophysiological processes (Lagace et al., 2010; Friedman et al., 2014). An understanding of the individual differences in microstructural hippocampal changes between those susceptible and resilient to stress will be useful for elaborating the neurobiological mechanisms under stress and for developing more targeted interventions.

Moreover, structural and functional disparities have been demonstrated between the dorsal (dHi) and ventral hippocampus (vHi), in which the dHi receives input information from cortical regions and plays a role in learning and spatial memory, while the vHi projects to the prefrontal cortex and is highly connected to the amygdala, bed nucleus of stria terminalis (BNST), and hypothalamic–pituitary–adrenal (HPA) axis, leading to its involvement in emotional regulation and behavioral motivation (Fanselow and Dong, 2010; Kheirbek and Hen, 2011; Bannerman et al., 2014; O’Leary and Cryan, 2014). Moreover, there is growing evidence for hemispheric lateralization in the hippocampus (Hou et al., 2013), as well as for asymmetric effects imposed by stress (Zach et al., 2016). Therefore, studies of the whole hippocampus may mask regional-specific stress effects (Nasca et al., 2017) and contribute to the heterogeneities observed in previous studies. Accordingly, the bilateral dHi and vHi should be investigated as separate brain regions.

Therefore, in the present study, we used a repeated DTI method to explore longitudinal microstructural alterations in the bilateral dHi and vHi of CSDS-susceptible and resilient mice to test the following hypotheses: (1) basic DTI indices are capable of being sensitive to microstructural alterations in hippocampal subregions under stress; (2) a pre-existing difference in subregional hippocampal microstructure between stress-susceptible and resilient mice may exist; (3) stress may induce distinct microstructural alterations in the two hemispheres of the hippocampus and along its dorso-ventral axis.

## Materials and Methods

### Animals

Adult male C57BL/6 mice (C57; 6–7 weeks old; Vital River Laboratories, Beijing, China) were housed in a group of five with free access to food and water and were acclimated to an average room temperature of 22°C with 50–60% humidity and proper day and night cycle from 8 AM to 8 PM for 1 week prior to experiments. Male CD-1 mice (10 weeks old; Vital River Laboratories) were singly housed and kept under the same conditions. This study was carried out in accordance with the National Institute of Health Guide for the Care and Use of Laboratory Animals. The protocol was approved by the Ethics Committee of Third Military Medical University.

### CSDS Protocol

The formal CSDS paradigm lasted for 10 days as described previously (Krishnan et al., 2007; Golden et al., 2011). Before the paradigm started, the CD-1 mice underwent a 3-day screening process. Once daily, a strange screener C57 mouse was placed directly into the home cage of the CD-1 mice for 3 min. A qualified CD-1 aggressor would attack in at least two consecutive sessions with a latency to initial aggression of less than 1 min. The chosen aggressors were then placed on the left side of adapted hamster cages (which were divided into two parts by a transparent perforated plexiglass) overnight before the formal initiation of the defeat sessions. At approximately 3:30 PM every afternoon for 10 consecutive days, each C57 mouse (*n* = 17) was first placed into a new aggressor’s home cage compartment for a 10-min social defeat session, followed by placement in the right side of the same cage for the remainder of the 24 h for continuous non-physical social stress exposure. On the other hand, the control C57 mice (*n* = 7) were housed with other C57 partners in the same adapted hamster cage, with one mouse on each side. The control mice were rotated to a new cage at the same time on a daily basis without any physical contact. After the last defeat session, all C57 mice (*n* = 24) were singly housed in standard mouse cages for at least 24 h before receiving the following behavior tests.

### Social Interaction Test (SIT)

**FIGURE 1:**
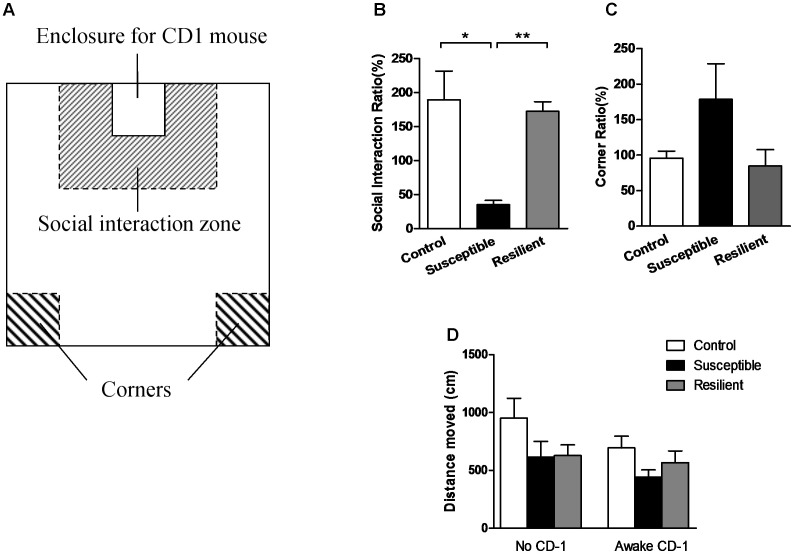
Social interaction test. **(A)** Schematic diagram of the social interaction arena. **(B)** Susceptible mice had a significantly lower SI ratio. **(C)** Susceptible mice had a non-significantly higher corner ratio. **(D)** Three groups displayed a comparable distance moved in the two separate trials. Data were presented as mean ± SEM; ^∗^*P* < 0.05, ^∗∗^*P* < 0.01.

Before the SIT was conducted, all experimental mice were placed in the laboratory room for acclimation for at least an hour. Then, according to the protocol (Golden et al., 2011), each C57 mouse was carefully placed in the center of the social field (**Figure 1A**) twice, either with an empty transparent perforated enclosure (first 150-s trial) or with a completely novel CD-1 mouse contained in the enclosure (second 150-s trial). Between the two trials, the C57 mice were placed back in their own cages for a 30-s rest period. The exploratory activity of the C57 mice in the two trials was recorded and analyzed by a video-tracking apparatus and software (Xinruan Information Technology Co., Shanghai, China). For each mouse, the social interaction (SI) ratio was calculated as (interaction time, CD-1 present)/(interaction time, CD-1 absent) × 100%, and the corner ratio was calculated as (corner time, CD-1 present)/(corner time, CD-1 absent) × 100%.

### *In vivo* DTI Data Acquisition and Processing

**FIGURE 2:**
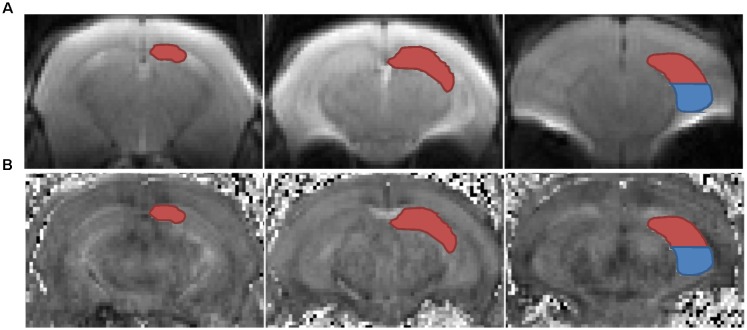
Left dorsal (red) and ventral (blue) hippocampus were delineated in representative coronal B_0_ maps **(A)** and FA maps **(B)**. ROIs, regions of interest.

To obtain longitudinal data, we conducted *in vivo* DTI scans twice, both before and after the CSDS paradigm, with a 7T magnetic resonance imaging (MRI) scanner (Bruker Bio Spec 70/20 USR). Experimental mice were initially anesthetized with 5% isoflurane, then placed on the scanning bed, and maintained with 1–2% isoflurane, and their respiration rate and body temperature were monitored throughout the DTI procedures. The portion of the brain between the olfactory bulb and the anterior cerebellum was scanned with the following parameters: TE = 25 ms, TR = 3000 ms, band width = 20000 Hz, slices = 10, slice thickness = 1 mm, b-value = 1000 s/mm^2^, diffusion directions = 30, gradient duration = 4.5 ms, gradient separation = 10.5 ms, image size = 128 × 128, FOV = 20 mm × 20 mm, and segments = 4. The data were processed using DTI-Studio software^1^. Motion correction and eddy current correction was performed by an automated image and registration package. The data were then interpolated to attain isotropic voxels and decoded to obtain the tensor field data, which were used to obtain the eigenvalues (λ_1_, λ_2_, λ_3_) and orthonormal eigenvectors (*e*1, *e*2, *e*3) and to compute the DTI indices with the following equations: $\mathrm{FA}=\sqrt{\frac{{\left(\lambda_{1}-\lambda_{2}\right)}^{2}+{\left(\lambda_{1}-\lambda_{3}\right)}^{2}+{\left(\lambda_{2}-\lambda_{3}\right)}^{2}}{2{\left(\lambda_{1}+\lambda_{2}+\lambda_{3}\right)}^{2}}}$; *MD* = ⅓ *Trace* = ⅓ (λ_1_ + λ_2_ + λ_3_); *RD* = ½ (λ_2_ + λ_3_); *AD* = λ_1_. Regions of interest (ROIs), including bilateral dHi and vHi, were manually drawn on the B_0_ images and the generated FA maps according to the mouse atlas (Paxinos and Franklin, 2001), Allen Mouse Brain Atlas^2^ (Lein et al., 2007) and previous studies (Kleinknecht et al., 2012; Reichel et al., 2017) by experimenters who were blind to group assignments (**Figure 2**). DTI indices were generated based on each ROI. Longitudinal percent change of DTI data was calculated as 100% × (post–pre)/pre. The degree of hemispheric asymmetry in hippocampal microstructure was also considered, which was calculated as the percentage difference between left and right ROIs’ diffusivity values: 100% × 2 × (left - right)/(left + right) (Madsen et al., 2012).

### Statistical Analyses

The statistical analysis was carried out using SPSS 18 (SPSS, Chicago, IL, United States) and illustrated with GraphPad Prism 5 (GraphPad Software, San Diego, CA, United States). Data were expressed as the mean ± SEM values. For the comparisons of the three groups (i.e., the susceptible, resilient and control groups), one-way analysis of variance (ANOVA) and two-sided Bonferroni *post hoc* test were used. Spearman’s correlation analysis was performed to examine the relationship between diffusivity values and SI ratios. The statistical significance level was set at *P* < 0.05.

## Results

### Social Interaction Test

The stressed mice were divided into two subgroups, with the susceptible mice (*n* = 10) defined as those with an SI ratio less than 100%, and the resilient mice (*n* = 7) defined as those with an SI ratio greater than 100%. The susceptible mice spent less time in the interaction zone or even hid in the corners when there was an aggressive CD-1 mouse present in the perforated enclosure, even though these mice had never previously interacted with this particular CD-1 mouse, giving them a significantly lower SI ratio [**Figure 1B**; *F*_(2, 21)_ = 15.000, *P* = 0.000; *post hoc* comparison: control vs. susceptible, *P* = 0.028; control vs. resilient, *P* = 0.973; susceptible vs. resilient, *P* = 0.000] and non-significantly higher corner ratio [**Figure 1C**; *F*_(2,21)_ = 1.897, *P* = 0.175] than the other two groups. Moreover, the three groups displayed a comparable distance moved in the two separate trials [**Figure 1D**; *F*_(2,21)_ = 0.545, *P* = 0.588], suggesting that the differences in social interaction had no correlation with the locomotory activity of the mice.

### Diffusion Properties of the Examined ROIs

Prior to the CSDS paradigm, a significantly lower FA value was found in the right vHi of the susceptible mice than in that of the resilient group [**Table 1**; *F*_(2, 21)_ = 3.896, *P* = 0.039; *post hoc* comparison: control vs. susceptible, *P* = 0.165; control vs. resilient, *P* = 0.998; susceptible vs. resilient, *P* = 0.047], suggesting that a pre-existing deficiency in the tissue integrity of the right vHi may be present in susceptible mice. However, there was no significant group difference in the FA values of any of the ROIs after stress exposure. In contrast, the MD (**Table 2**) and RD values (**Table 3**) presented a similar trend to one another; there was no significant difference in the MD or RD values of any ROI prior to stress, but after stress exposure, the resilient mice presented a higher MD and RD value in the right dHi than susceptible mice [MD: *F*_(2, 21)_ = 3.800, *P* = 0.045; *post hoc* comparison: control vs. susceptible, *P* = 0.999, control vs. resilient, *P* = 0.805, susceptible vs. resilient, *P* = 0.042, RD: *F*_(2,21)_ = 7.164, *P* = 0.006; *post hoc* comparison: control vs. susceptible, *P* = 0.618, control vs. resilient, *P* = 0.434, susceptible vs. resilient, *P* = 0.005], suggesting that a unique alteration may occur in the right dHi of resilient mice. However, there was no significant difference in AD values in either the pre- or post-stress comparisons (**Table 4**).

**Table 1:** Group comparisons of pre- and post-stress hippocampal FA values.

ROIs	Pre-stress					Post-stress				
	Control	Susceptible	Resilient	*F*_(2,21)_	*P*	Control	Susceptible	Resilient	*F*_(2,21)_	*P*
Left	0.499 ± 0.004	0.455 ± 0.043	0.507 ± 0.006	0.689	0.515	0.491 ± 0.023	0.487 ± 0.016	0.513 ± 0.049	0.205	0.817
L-dHi	0.498 ± 0.003	0.499 ± 0.008	0.506 ± 0.005	0.261	0.773	0.488 ± 0.026	0.486 ± 0.014	0.509 ± 0.050	0.165	0.849
L-vHi	0.502 ± 0.007	0.487 ± 0.008	0.503 ± 0.009	1.094	0.356	0.450 ± 0.045	0.481 ± 0.020	0.500 ± 0.052	0.314	0.735
Right	0.508 ± 0.009	0.496 ± 0.010	0.505 ± 0.008	0.482	0.625	0.484 ± 0.029	0.482 ± 0.014	0.517 ± 0.045	0.468	0.635
R-dHi	0.512 ± 0.012	0.496 ± 0.010	0.505 ± 0.005	0.557	0.583	0.481 ± 0.028	0.473 ± 0.013	0.513 ± 0.045	0.600	0.561
R-vHi	0.510 ± 0.008	0.470 ± 0.013	0.511 ± 0.009	3.896	0.039^∗^	0.496 ± 0.053	0.456 ± 0.029	0.522 ± 0.048	0.831	0.454

*Data were presented as mean ± SEM. ^∗^P < 0.05; ^∗∗^P < 0.01. dHi, dorsal hippocampus; FA, fractional anisotropy; ROIs, regions of interest; vHi, ventral hippocampus.*

**Table 2:** Group comparisons of pre- and post-stress hippocampal MD values (10^−4^mm^2^/s).

ROIs	Pre-stress					Post-stress				
	Control	Susceptible	Resilient	*F*_(2,21)_	*P*	Control	Susceptible	Resilient	*F*_(2,21)_	*P*
Left	5.454 ± 0.096	5.441 ± 0.069	5.512 ± 0.160	0.121	0.886	2.896 ± 0.102	2.756 ± 0.122	3.206 ± 0.249	1.860	0.188
L-dHi	5.474 ± 0.099	5.426 ± 0.096	5.567 ± 0.189	0.303	0.742	2.848 ± 0.058	2.762 ± 0.121	3.199 ± 0.299	1.455	0.263
L-vHi	5.408 ± 0.234	5.633 ± 0.096	5.453 ± 0.153	0.755	0.484	2.982 ± 0.118	2.797 ± 0.126	2.917 ± 0.191	0.318	0.732
Right	5.488 ± 0.203	5.555 ± 0.075	5.689 ± 0.150	0.563	0.579	2.914 ± 0.095	2.799 ± 0.129	2.999 ± 0.162	0.527	0.600
R-dHi	5.505 ± 0.173	5.512 ± 0.079	5.478 ± 0.162	0.022	0.979	2.913 ± 0.091	2.708 ± 0.097	3.186 ± 0.174	3.800	0.045^∗^
R-vHi	5.566 ± 0.272	5.998 ± 0.240	6.201 ± 0.207	1.175	0.331	3.201 ± 0.176	2.843 ± 0.146	3.076 ± 0.173	1.033	0.379

*Data were presented as mean ± SEM. ^∗^P < 0.05; ^∗∗^P < 0.01. dHi, dorsal hippocampus; MD, mean diffusivity; ROIs, regions of interest; vHi, ventral hippocampus.*

**Table 3:** Group comparisons of pre- and post-stress hippocampal RD values (10^−4^mm^2^/s).

ROIs	Pre-stress					Post-stress				
	Control	Susceptible	Resilient	*F*_(2,21)_	*P*	Control	Susceptible	Resilient	*F*_(2,21)_	*P*
Left	4.320 ± 0.075	4.232 ± 0.060	4.313 ± 0.113	0.357	0.705	2.237 ± 0.014	2.140 ± 0.075	2.508 ± 0.188	2.618	0.104
L-dHi	4.333 ± 0.076	4.204 ± 0.074	4.347 ± 0.014	0.651	0.534	2.211 ± 0.027	2.146 ± 0.078	2.516 ± 0.237	1.892	0.183
L-vHi	4.285 ± 0.167	4.433 ± 0.066	4.278 ± 0.094	0.998	0.388	2.404 ± 0.083	2.199 ± 0.071	2.309 ± 0.112	1.037	0.377
Right	4.271 ± 0.180	4.309 ± 0.055	4.410 ± 0.110	0.482	0.625	2.277 ± 0.047	2.184 ± 0.084	2.297 ± 0.061	0.568	0.578
R-dHi	4.253 ± 0.163	4.277 ± 0.072	4.292 ± 0.126	0.023	0.977	2.291 ± 0.050	2.117 ± 0.060	2.510 ± 0.103	7.164	0.006^∗∗^
R-vHi	4.329 ± 0.242	4.742 ± 0.176	4.725 ± 0.173	1.019	0.381	2.508 ± 0.152	2.247 ± 0.085	2.422 ± 0.136	1.262	0.310

*Data were presented as mean ± SEM. ^∗^P < 0.05; ^∗∗^P < 0.01. dHi, dorsal hippocampus; RD, radial diffusivity; ROIs, regions of interest; vHi, ventral hippocampus.*

**Table 4:** Group comparisons of pre- and post-stress hippocampal AD values (10^−4^mm^2^/s).

ROIs	Pre-stress					Post-stress				
	Control	Susceptible	Resilient	*F*_(2,21)_	*P*	Control	Susceptible	Resilient	*F*_(2,21)_	*P*
Left	7.720 ± 0.139	7.859 ± 0.144	7.911 ± 0.262	0.170	0.845	4.216 ± 0.290	3.990 ± 0.227	4.602 ± 0.407	1.112	0.353
L-dHi	7.755 ± 0.146	7.869 ± 0.190	8.007 ± 0.296	0.219	0.806	4.121 ± 0.220	3.992 ± 0.219	4.564 ± 0.458	0.916	0.420
L-vHi	7.651 ± 0.375	8.032 ± 0.185	7.801 ± 0.287	0.529	0.598	4.140 ± 0.291	3.991 ± 0.248	4.131 ± 0.370	0.076	0.927
Right	7.923 ± 0.264	8.048 ± 0.170	8.247 ± 0.242	0.462	0.637	4.189 ± 0.285	4.027 ± 0.231	4.403 ± 0.365	0.457	0.641
R-dHi	8.008 ± 0.219	7.980 ± 0.175	7.850 ± 0.237	0.144	0.867	4.156 ± 0.245	3.889 ± 0.182	4.537 ± 0.351	1.786	0.200
R-vHi	8.040 ± 0.356	8.509 ± 0.396	9.152 ± 0.304	1.588	0.232	4.586 ± 0.385	4.036 ± 0.279	4.385 ± 0.324	0.651	0.535

*Data were presented as mean ± SEM. ^∗^P < 0.05; ^∗∗^P < 0.01. AD, axial diffusivity; dHi, dorsal hippocampus; ROIs, regions of interest; vHi, ventral hippocampus.*

In the longitudinal comparisons (**Supplementary Table S1**), a significant difference was only found in the RD value of the right dHi, which decreased in the susceptible group more than in the resilient group [control = −47.67 ± 0.58%, susceptible = −50.45 ± 1.42%, resilient = −41.01 ± 3.32%; *F*_(2, 21)_ = 5.225, *P* = 0.018; *post hoc* comparison: control vs. susceptible, *P* = 0.999, control vs. resilient, *P* = 0.349, susceptible vs. resilient, *P* = 0. 016].

### Associations Between the SI Ratio and Diffusion Properties

Correlation analysis (**Supplementary Table S2**) revealed a significant positive relationship between the SI ratio and post-stress left MD (*r*_24_ = 0.497, *P* = 0.031), right dHi MD (*r*_24_ = 0.507, *P* = 0.027), and right dHi RD values (*r*_24_ = 0.594, *P* = 0.007), as well as the percent change in the longitudinal right dHi MD (*r*_24_ = 0.504, *P* = 0.028), suggesting that the microstructural alterations reflected by these regional DTI indices may contribute some protection associated with stress resilience. However, no significant correlations were found between the SI ratio and any pre-stress DTI indices.

To test whether the stress paradigm imposed an asymmetric effect on the hippocampus, the associations between the SI ratio and the hippocampal bilateral mean of the four DTI indices were analyzed. A negative relationship was found between the SI ratio and post-stress left relative to right vHi FA (**Supplementary Table S2**; *r*_24_ = −0.570, *P* = 0.011). That is, the higher the post-stress right relative to left vHi FA, the higher the SI ratio and the greater the stress resilience, suggesting a protective role of the right ventral hippocampal tissue integrity under stress.

## Discussion

To examine whether there are hippocampal microstructural alterations underlying chronic social stress susceptibility, we used a longitudinal *in vivo* DTI method to document the changes in hippocampal diffusion pattern in a CSDS model using basic DTI indices, aiming to contribute to the understanding of the involved causality. Meanwhile, we also investigated whether there are asymmetric effects imposed by stress between the left and right as well as the dorsal and ventral portions of the hippocampus, which may be of therapeutic and preventive value for stress-related psychiatric disorders.

Consistent with our hypothesis, prior to CSDS, a significantly lower FA value was found in the right vHi of the susceptible mice compared to that of the resilient group, which may have contributed to the division of the following behavioral response to stress. The FA value reflects cell number, tissue density, and cell structure alignment; therefore, this pre-existing lower FA value may result from an inconsistent orientation, reduced cell structure alignment, decreased myelination or increased interference from neuroinflammation, gliosis or other factors that limit the diffusion of water molecules in the right vHi of the susceptible mice (Beppu et al., 2005; Alexander et al., 2007; Kinoshita et al., 2008; Evans, 2013; Kumar et al., 2014; Anacker et al., 2016). Concerning cellular changes have been demonstrated postmortem in the hippocampus of major depressive disorder (MDD) (Stockmeier et al., 2004) and PTSD patients (Krystal and Duman, 2004). However, due to the paucity of longitudinal studies, including pre-stress hippocampal DTI data, these assumptions still need to be verified with further research combined with other methods.

No significant group differences were found in the post-stress FA values of any of the hippocampal ROIs examined, nor were any significant correlations between SI ratios and pre-stress, post-stress or longitudinal changes in FA values observed, which is consistent with previous negative results (Delgado y Palacios et al., 2011; Kumar et al., 2014; Khan et al., 2016). However, Anacker et al. (2016), with an *ex vivo* MRI method, revealed that the hippocampal FA value of CSDS mice were positively correlated with their social avoidance index, suggesting that water diffusion is more anisotropic in stress-susceptible mice. This disparity may be due to the differences between the *in vivo* and *ex vivo* experimental conditions, making the results difficult to compare directly (Khan et al., 2016). Moreover, the correlation was not significant in the independently analyzed control group in the *ex vivo* study; therefore, an analysis including all three groups may reveal that the mild difference observed in the stressed groups is no longer significant. On the other hand, in a recent clinical study (Srivastava et al., 2016), a significantly lower FA value was found in the right hippocampus of patients with first-episode, treatment-naive MDD than that found in the right hippocampus of healthy controls and was believed to potentially reflect the decrease in hippocampal volume of MDD patients found in previous studies (Frodl et al., 2002; Malykhin et al., 2010). Therefore, macrostructural volume changes in the hippocampus may be reflected by alterations in its microstructure.

In addition, according to the mathematical definition of the FA value, its interpretation needs to be combined with MD, RD and AD values. No significant differences were observed in pre-stress MD, RD, or AD values in any of the hippocampal ROIs examined among the three groups. After exposure to the CSDS paradigm, a significant increase in MD and RD values was only observed in the right dHi of the resilient group, indicating that a unique change may occur in this region of the resilient brain. The MD value in the hippocampus is generally considered to be closely related to hippocampal function. For example, in healthy individuals, people with higher education (Piras et al., 2011) or better memory function (Carlesimo et al., 2010) have been demonstrated to have a lower hippocampal MD value. Higher MD values may result from reduced membrane density and may also reflect neuronal arborization or increased extracellular space (Londono et al., 2003; Kumar et al., 2014). Meanwhile, a higher RD value in white matter is usually associated with demyelination or dysmyelination (Song et al., 2002). In the hippocampus, however, there are two other possible explanations for an increase in RD: dendritic atrophy and loss of neurites may lead to a decrease in directional diffusion of water molecules, or augmented arborization may increase the diffusion directions along dendrites (Delgado y Palacios et al., 2014). Both the concerning postmortem examinations (Stockmeier et al., 2004) and studies conducted in various animal models of stress seem to support the latter possibility. For example, reduced interneuronal dendritic arborization in the hippocampal CA1 region was found in chronic mild restraint-stressed mice (Gilabert-Juan et al., 2017). Furthermore, chronic psychosocial stress was shown to result in decreased astroglial plasticity in the hippocampus (Czeh et al., 2006), and the density of dendritic spines in the hippocampus of susceptible mice was found to be significantly lower than in the hippocampus of control and stress-resilient mice in the learned helplessness (Yang et al., 2015) and chronic mild stress (CMS) (Li et al., 2011, 2014) paradigms. In addition, direct evidence of plastic changes has been shown to occur in the hippocampus of stress-resilient subgroups, including neurogenesis in the dentate gyrus and dendrite and synapse remodeling in major neurons of Ammon’s horn (Li et al., 2014; McEwen et al., 2015). Moreover, from the perspective of treatment, anti-depressants, such as imipramine, have been shown to prevent depressive-like behaviors through increasing dendritic branching of immature neurons (Fenton et al., 2015) and contribute to resilience to CMS re-exposure by re-establishing hippocampal neurogenesis and neuronal dendritic arborization (Alves et al., 2017). Although further studies are needed to shed light on the underlying mechanisms of the diffusion changes observed in the right hippocampus of resilient individuals, the increased MD and RD values observed in the resilient mice in the present study may result from augmented dendritic arborization and neuronal plasticity.

In the examination of the AD value, no significant group differences or longitudinal changes were found in the hippocampus, which was also negative in another study of CMS paradigm (Delgado y Palacios et al., 2011). However, a decreased AD value, not FA, MD or RD, was detected in the left hippocampus of mice exposed to CMS compared to that in the left hippocampus of controls, which indicated a destruction of neurofibrils (Kumar et al., 2014). The inconsistency of these results could be attributed to the different stress paradigms that were applied. The lack of a differentiation between susceptible animals and those that are resilient may have also masked subtle hippocampal changes in stress-induced subgroups. Moreover, in a recent study of veterans (Waltzman et al., 2017), the right hippocampal AD value was significantly higher in the PTSD group than in the traumatic brain injury group; however, compared to that of the control group, there were no significant differences in the bilateral hippocampal DTI indices of those individuals with PTSD. This finding demonstrated that no significant axonal injury in the hippocampus of PTSD patients was detected by DTI, which is consistent with our findings.

Our findings also revealed an asymmetric effect induced by stress on microstructural changes in hippocampal subregions, emphasizing the role of the right hippocampus in the underlying mechanisms of stress-related disorders. Glucocorticoids may cause the lateral effect of stress on the hippocampus, which has been predominantly observed in the right side (Tang et al., 2008; Zach et al., 2010, 2016; Frodl and O’Keane, 2012). However, higher HPA axis activation (basal cortisol levels) has been found to be associated with higher left relative to right hippocampal MD, suggesting that either the left hippocampus plays a lateralized regulatory role in HPA axis function or the neuroendocrine response under stress imposes left-sided effects on hippocampal microstructure (Madsen et al., 2012). This asymmetric role of the bilateral hippocampus in stress may also be due to asymmetries in neuronal numbers and neurogenesis, as well as proteomics and genomics (Hou et al., 2013). Further studies are required to examine the molecular mechanisms underlying such asymmetry.

Our investigation of the dorsal and ventral portions of the hippocampus suggested that the difference in ventral hippocampal microstructure seemed to act as a pre-existing factor in the following behavioral phenotypes, whereas the dHi was more influenced by the stress paradigm and demonstrated adaptive changes. Structural and functional differences in the dHi and vHi have been put forward previously (Fanselow and Dong, 2010), and their specific role under stress has drawn an increasing amount of attention. For example, after chronic unpredictable stress (CUS), a volumetric reduction and increase was revealed in the dHi and vHi, respectively, concurrent with dendritic atrophy and enrichment in the respective hippocampal subregions (Pinto et al., 2015). The vHi is usually believed to be more involved in emotional regulation and the stress response (Trivedi and Coover, 2004; Adami et al., 2006; Pentkowski et al., 2006; Kenworthy et al., 2014) through neurogenesis (O’Leary et al., 2012; Tanti et al., 2012) and epigenetics (Nasca et al., 2017). In the present study, the significantly lower pre-stress FA value also exhibited in the vHi. However, after stress, a greater microstructural alteration was demonstrated in the dHi than in the vHi, especially in the resilient group. Consistent with our findings, an analysis of the change in the number of hippocampal interneurons of specific subtypes after CMS revealed a more pronounced effect on the dHi than vHi (Czeh et al., 2015). Moreover, CUS selectively decreased cell survival in the vHi, while it induced adaptive neuroplasticity primarily in the dHi, which was closely related to the following behavioral responses, such as avoidance or amelioration of the stressor (Hawley and Leasure, 2012). But whether the pre-existed FA differences in the right vHi drive the changes in dHi MD/RD values in response to stress is still need further longitudinal studies based on the specific role of the vHi and dHi both before and after stress.

The present study had some limitations. First, our study lacked a direct observation of neuron and dendrite density in the bilateral hippocampus of stress-susceptible and resilient mice using immunohistological methods, which would be necessary for a more in-depth explanation of the longitudinal changes of diffusion properties. Second, this longitudinal work did not examine other stress-related brain regions. Covariant microstructural changes might be more closely related to the mechanisms underlying stress-related psychiatry or stress resilience. In addition, since the main focus of the current study was to investigate the longitudinal microstructural changes in the bilateral hippocampus in different stress phenotypes, only an ROI-based method was used rather than a voxel-based analysis or tract-based spatial statistics. Third, more hippocampal-dependent function tasks might be added to test whether there is a corresponding change in memory and learning, contextual fear conditioning, or other hippocampus-dependent cognitional or emotional regulations associated with the detected microstructural changes. Moreover, echo-planar imaging (EPI) sequence was chosen over turbo spin echo (TSE) in this study for its common use, less imaging time and motion artifact (Poustchi-Amin et al., 2001), however, taking the image quality into consideration, TSE could be a good alternative (Hirata et al., 2018). Finally, due to the limited sample size, some more subtle differences may have not reached a significant level. In conclusion, this non-invasive *in vivo* DTI method allowed us to detect subtle microstructural alterations in the bilateral dHi and vHi of different stress phenotypes in a CSDS mouse model. Our data revealed a lower FA value in the right vHi of the susceptible mice than in the right vHi of resilient mice that was present prior to stress, suggesting that pre-existing microstructural abnormalities may result in stress susceptibility. Meanwhile, increases in MD and RD values were found in the right dHi of the resilient subgroup after stress compared to those before stress, which may indicate that plastic changes that prevent individuals from exhibiting social avoidance behavior occur in response to stress. Furthermore, the asymmetric effect imposed by stress highlights the important role of the right hippocampus, which may be the key to understanding the mechanism underlying stress-related disorders as well as become a potential target for resilience training or more effective interventions.

## Author Contributions

XL conducted the experiments, analyzed the data, and drafted the manuscript. ZF designed the experiments. JY and YG performed some behavioral tests. YG, XW, and ZF revised the paper.

## Conflict of Interest Statement

The authors declare that the research was conducted in the absence of any commercial or financial relationships that could be construed as a potential conflict of interest.

## Footnotes

1 http://lbam.med.jhmi.edu/

2 http://atlas.brain-map.org/
