# Supplementary material for: Longitudinal in vivo Diffusion Tensor Imaging Detects Differential Microstructural Alterations in the Hippocampus of Chronic Social Defeat Stress-Susceptible and Resilient Mice
Source: Front Neurosci. 2018 Aug 29;12:613. doi: 10.3389/fnins.2018.00613 (PMC6123364; doi:10.3389/fnins.2018.00613)
Supplement: Supplementary file 1 [file Table_1.DOCX]

**Supplementary Material**

**TABLE S1**｜**Group comparisons of longitudinal changes(%) of DTI indices**

| ROIs | Groups | FA | |  | MD | |  | RD | |  | AD | |
| --- | --- | --- | --- | --- | --- | --- | --- | --- | --- | --- | --- | --- |
|  |  | mean±SEM | *P* |  | mean±SEM | *P* |  | mean±SEM | *P* |  | mean±SEM | *P* |
| Left | C | -1.05±4.36 | 0.979 |  | -47.09±3.10 | 0.177 |  | -48.53±1.30 | 0.060 |  | -45.45±5.14 | 0.401 |
|  | S | -0.80±4.26 |  |  | -49.36±2.11 |  |  | -49.49±1.44 |  |  | -49.10±2.99 |  |
|  | R | 0.96±10.62 |  |  | -41.71±3.90 |  |  | -41.58±3.57 |  |  | -41.78±4.87 |  |
| L-dHi | C | -1.34±4.84 | 0.953 |  | -48.29±2.22 | 0.284 |  | -49.34±0.55 | 0.129 |  | -47.11±4.10 | 0.533 |
|  | S | -2.35±3.66 |  |  | -49.03±2.24 |  |  | -48.93±1.72 |  |  | -49.00±3.00 |  |
|  | R | 0.28±9.98 |  |  | 42.53±4.05 |  |  | -42.02±3.76 |  |  | -43.05±4.90 |  |
| L-vHi | C | -9.06±9.10 | 0.812 |  | -44.20±4.98 | 0.429 |  | -43.86±2.63 | 0.185 |  | -44.49±7.75 | 0.671 |
|  | S | -1.35±3.80 |  |  | -50.28±2.18 |  |  | -50.25±1.85 |  |  | -50.32±2.73 |  |
|  | R | -0.13±12.65 |  |  | 46.48±3.66 |  |  | -45.91±2.56 |  |  | -46.94±5.27 |  |
| Right | C | -3.39±6.80 | 0.828 |  | -48.23±3.09 | 0.803 |  | -48.55±0.91 | 0.807 |  | -47.82±5.51 | 0.827 |
|  | S | -2.21±3.74 |  |  | -49.56±2.35 |  |  | -49.33±1.77 |  |  | -49.71±3.08 |  |
|  | R | 2.48±9.73 |  |  | -47.14±2.89 |  |  | -48.67±1.75 |  |  | -46.55±4.37 |  |
| R-dHi | C | -5.08±7.50 | 0.751 |  | -48.36±1.98 | 0.064 |  | -47.67±0.57 | **0.018*** |  | -49.00±4.15 | 0.208 |
|  | S | -4.23±3.61 |  |  | -50.86±1.83 |  |  | -50.45±1.41 |  |  | -51.02±2.52 |  |
|  | R | 1.51±9.13 |  |  | -41.36±4.08 |  |  | -41.01±3.32 |  |  | -41.76±5.25 |  |
| R-vHi | C | -1.27±10.58 | 0.876 |  | -42.86±5.18 | 0.384 |  | -43.46±3.12 | 0.350 |  | -42.21±7.86 | 0.452 |
|  | S | -2.68±6.02 |  |  | -51.75±3.38 |  |  | -51.80±2.96 |  |  | -51.68±3.96 |  |
|  | R | 3.20±11.48 |  |  | -49.78±2.89 |  |  | -47.99±3.66 |  |  | -51.44±3.43 |  |

Data were presented as mean±SEM. *: *P*﹤0.05. FA=fractional anisotropy; MD=mean diffusivity; RD=radial diffusivity; AD=axial diffusivity; ROIs=regions of interest; dHi=dorsal hippocampus; vHi=ventral hippocampus.
