# Supplementary material for: Longitudinal in vivo Diffusion Tensor Imaging Detects Differential Microstructural Alterations in the Hippocampus of Chronic Social Defeat Stress-Susceptible and Resilient Mice
Source: Front Neurosci. 2018 Aug 29;12:613. doi: 10.3389/fnins.2018.00613 (PMC6123364; doi:10.3389/fnins.2018.00613)
Supplement: Supplementary file 2 [file Table_2.DOCX]

**Supplementary Material**

**TABLE S2**｜ **Associations between the SI ratio and diffusion properties**

| ROIs | | SI% | | | | | | | |
| --- | --- | --- | --- | --- | --- | --- | --- | --- | --- |
|  |  | FA | | MD | | RD | | AD | |
|  |  | r | *P* | r | *P* | r | *P* | r | *P* |
| pre-stress | Left | 0.098 | 0.673 | 0.066 | 0.775 | 0.205 | 0.374 | -0.101 | 0.662 |
|  | L-dHi | -0.177 | 0.443 | 0.119 | 0.608 | 0.177 | 0.444 | -0.055 | 0.812 |
|  | L-vHi | 0.139 | 0.547 | -0.286 | 0.209 | -0.286 | 0.209 | -0.123 | 0.596 |
|  | Right | 0.071 | 0.758 | 0.103 | 0.658 | 0.155 | 0.501 | 0.016 | 0.944 |
|  | R-dHi | 0.227 | 0.321 | -0.055 | 0.814 | -0.040 | 0.865 | -0.153 | 0.507 |
|  | R-vHi | 0.402 | 0.071 | -0.306 | 0.177 | -0.309 | 0.173 | -0.029 | 0.900 |
|  |  |  |  |  |  |  |  |  |  |
| post-stress | **Left** | -0.090 | 0.716 | **0.497** | **0.031*** | 0.453 | 0.052 | 0.301 | 0.210 |
|  | L-dHi | -0.094 | 0.701 | 0.430 | 0.066 | 0.423 | 0.071 | 0.214 | 0.379 |
|  | L-vHi | -0.251 | 0.300 | 0.206 | 0.397 | 0.279 | 0.247 | 0.059 | 0.811 |
|  | Right | -0.095 | 0.699 | 0.309 | 0.198 | 0.455 | 0.051 | 0.276 | 0.252 |
|  | **R-dHi** | 0.006 | 0.980 | **0.507** | **0.027*** | **0.594** | **0.007**** | 0.400 | 0.090 |
|  | R-vHi | 0.180 | 0.461 | 0.151 | 0.537 | 0.140 | 0.569 | 0.171 | 0.484 |
|  |  |  |  |  |  |  |  |  |  |
| longitudinal change% | Left | -0.214 | 0.379 | 0.452 | 0.052 | 0.453 | 0.052 | 0.419 | 0.074 |
|  | L-dHi | -0.033 | 0.892 | 0.376 | 0.112 | 0.338 | 0.157 | 0.308 | 0.199 |
|  | L-vHi | -0.348 | 0.144 | 0.317 | 0.186 | 0.401 | 0.089 | 0.069 | 0.778 |
|  | Right | -0.039 | 0.872 | 0.373 | 0.116 | 0.344 | 0.149 | 0.305 | 0.205 |
|  | **R-dHi** | 0.066 | 0.789 | **0.504** | **0.028*** | 0.433 | 0.064 | 0.428 | 0.067 |
|  | R-vHi | -0.047 | 0.850 | 0.198 | 0.416 | 0.254 | 0.293 | 0.216 | 0.375 |
|  |  |  |  |  |  |  |  |  |  |
| bilateral mean | pre-Hi | -0.092 | 0.708 | -0.029 | 0.906 | 0.223 | 0.359 | -0.219 | 0.367 |
|  | pre-dHi | -0.348 | 0.144 | 0.216 | 0.375 | 0.210 | 0.389 | 0.011 | 0.966 |
|  | pre-vHi | -0.216 | 0.375 | 0.052 | 0.833 | 0.162 | 0.507 | -0.064 | 0.794 |
|  | post-Hi | -0.079 | 0.748 | 0.196 | 0.422 | 0.346 | 0.147 | 0.172 | 0.481 |
|  | post-dHi | -0.258 | 0.286 | -0.234 | 0.334 | -0.253 | 0.297 | -0.159 | 0.516 |
|  | **post-vHi** | **-0.570** | **0.011** | -0.135 | 0.581 | -0.011 | 0.963 | -0.156 | 0.523 |

*: *P*﹤0.05; **: *P*﹤0.01. SI=social interaction; FA=fractional anisotropy; MD=mean diffusivity; RD=radial diffusivity; AD=axial diffusivity; ROIs=regions of interest; dHi=dorsal hippocampus; vHi=ventral hippocampus.
